# Second Report of Chronic Granulomatous Disease in Jordan: Clinical and Genetic Description of 31 Patients From 21 Different Families, Including Families From Lybia and Iraq

**DOI:** 10.3389/fimmu.2021.639226

**Published:** 2021-03-05

**Authors:** Faris Ghalib Bakri, Michelle Mollin, Sylvain Beaumel, Bénédicte Vigne, Nathalie Roux-Buisson, Adel Mohammed Al-Wahadneh, Raed Mohammed Alzyoud, Wail Ahmad Hayajneh, Ammar Khaled Daoud, Mohammed Elian Abu Shukair, Mansour Fuad Karadshe, Mahmoud Mohammad Sarhan, Jamal Ahmad Wadi Al-Ramahi, Julien Fauré, John Rendu, Marie Jose Stasia

**Affiliations:** ^1^Division of Infectious Diseases, Department of Medicine, Jordan University Hospital, Amman, Jordan; ^2^Infectious Diseases and Vaccine Center, University of Jordan, Amman, Jordan; ^3^Centre Hospitalier Universitaire Grenoble Alpes, Pôle de Biologie, Chronic Granulomatous Disease Diagnosis and Research Centre (CDiReC), Grenoble, France; ^4^Centre Hospitalier Universitaire Grenoble Alpes, Pôle de Biologie, Laboratoire de Biochimie et Génétique Moléculaire, La Tronche, France; ^5^Université Grenoble Alpes, Inserm U1216, Grenoble Institut Neurosciences, Grenoble, France; ^6^Division of Immunology, Department of Pediatrics, Queen Rani Children's Hospital, Amman, Jordan; ^7^Division of Infectious Diseases, Department of Pediatrics, Jordan University of Science & Technology, Irbid, Jordan; ^8^Division of Immunology, Jordan University of Science & Technology, Irbid, Jordan; ^9^Jo PAIR clinic, Amman, Jordan; ^10^Josante Medical Center, Amman, Jordan; ^11^Sarhan Oncology and Stem Cell Clinics, Amman, Jordan; ^12^Adjunct Faculty, Jordan University Hospital, Amman, Jordan; ^13^Université Grenoble Alpes, Commissariat à l'Energie Atomique (CEA), Centre National de la Recherche Scientifique (CNRS), Institut de Biologie Structurale (IBS), Grenoble, France

**Keywords:** innate immunodeficiency, Jordan, chronic granulomatous disease, autosomal recessive, NADPH oxidase, founder mutation

## Abstract

Chronic granulomatous Disease (CGD) is a rare innate immunodeficiency disorder caused by mutations in one of the six genes (*CYBA, CYBB, NCF1, NCF2, NCF4*, and *CYBC1*/EROS) encoding the superoxide-producing nicotinamide adenine dinucleotide phosphate (NADPH)—oxidase complex in phagocytes. In the Western population, the most prevalent form of CGD (about two-thirds of all cases) is the X-linked form (X-CGD) caused by mutations in *CYBB*. The autosomal recessive forms (AR-CGD), due to mutations in the other genes, collectively account for the remaining one-third of CGD cases. We investigated the clinical and molecular features of 22 Jordanian, 7 Libyan, and 2 Iraqi CGD patients from 21 different families. In addition, 11 sibling patients from these families were suspected to have been died from CGD as suggested by their familial and clinical history. All patients except 9 were children of consanguineous parents. Most of the patients suffered from AR-CGD, with mutations in *CYBA, NCF1*, and *NCF2*, encoding p22^*phox*^, p47^*phox*^, and p67^*phox*^ proteins, respectively. AR-CGD was the most frequent form, in Jordan probably because consanguineous marriages are common in this country. Only one patient from non-consanguineous parents suffered from an X91^0^ CGD subtype (0 indicates no protein expression). AR67^0^ CGD and AR22^0^ CGD appeared to be the most frequently found sub-types but also the most severe clinical forms compared to AR47^0^ CGD. As a geographical clustering of 11 patients from eight Jordanian families exhibited the c.1171_1175delAAGCT mutation in *NCF2*, segregation analysis with nine polymorphic markers overlapping *NCF2* indicates that a common ancestor has arisen ~1,075 years ago.

## Introduction

Chronic granulomatous disease (CGD) is a rare congenital immunodeficiency syndrome with an incidence of 1 in ~250,000 individuals. CGD is a genetically heterogeneous disease with all ethnic groups equally affected. The disease results from defects in one of the five components of the nicotinamide adenine dinucleotide phosphate (NADPH) oxidase complex in phagocytes, i.e., the membrane proteins gp91^*phox*^ (or NOX2) and p22^*phox*^ (together forming cytochrome *b*_558_) and the cytosolic components p47^*phox*^, p67^*phox*^, and p40^*phox*^ (1). These proteins are present in phagocytic leukocytes (neutrophils, eosinophils, monocytes, and macrophages). In addition, it was recently demonstrated that mutations in EROS/*CYBC1* are responsible for a decrease in NADPH oxidase activity of phagocytes and could lead to the development of CGD (2).

In resting phagocytes, the NADPH oxidase enzyme is dissociated and becomes complexed upon stimulation by specific interactions of opsonized microorganisms with membrane receptors. When the enzyme is assembled, the gp91^*phox*^ flavin- and heme-containing oxidase element becomes capable of transferring electrons from NADPH in the cytosol to molecular oxygen in the extracellular or intra-phagosomal compartment, while the p22^*phox*^ subunit stabilizes the expression of gp91^*phox*^ in phagocytic cells (3). The NADPH oxidase complex catalyzes the conversion of molecular oxygen O_2_ to superoxide anion ${\text{O}}_{2}^{-}$. Thus, this enzyme is the key of the generation of toxic reactive oxygen species responsible for the intracellular killing of microorganisms. As a result, CGD patients, with a defect in this system, suffer from recurrent and often life-threatening bacterial and fungal infections (4, 5). In addition, inflammatory manifestations are common in CGD and are often associated with neutrophil and/or granulomatous inflammation. These inflammatory diseases affect the gastrointestinal tract, lung, skin, and genitourinary tract and can cause overt autoimmune disease. Gastrointestinal manifestations or lung granuloma of CGD can precede the onset of infectious symptoms and can mimic symptoms of Crohn's disease or sarcoidosis, respectively (6).

The most frequently found form of CGD is the X-linked form, with mutations in the *CYBB* gene encoding gp91^*phox*^ subunit or NOX2 (~70% of CGD cases). The AR-CGD form most frequently encountered is due to mutations in *NCF1* encoding p47^*phox*^ (accounting for ~25% of CGD cases). The often-reported recurrent mutation ΔGT at the beginning of exon 2 is caused by recombination events between *NCF1* and two pseudogenes surrounding *NCF1* (7). Rare AR subgroups (<5% of CGD cases) are caused by mutations in *CYBA, NCF2* or *NCF4* genes encoding p22^*phox*^, p67^*phox*^, or p40^*phox*^ subunits, respectively (8–10). In very rare cases, the small G protein, Rac2, involved in regulating NADPH oxidase activity could also be mutated leading to a specific innate immunodeficiency characterized by an unresponsiveness to the chemotactic peptide FormylMetLeuPhe (for NADPH oxidase activity and chemotaxis) (11–15).

Based on several cohort studies, it appears that the X-CGD form affecting the membrane redox element of the NADPH oxidase complex is related to the most severe clinical features (4, 5, 16, 17). However, it is important to note that mutations in NADPH oxidase genes leading to residual activity, such as certain X^−^-CGDvariants or some *NCF1* mutations appear to be associated with milder clinical forms (16, 18–20). One way to obtain information on the severity of rare cases of AR67^0^ and AR22^0^CGD is to investigate patients in countries in which these forms predominate. We have previously reported for the first time the clinical and genetic investigations of 15 Jordanian patients from nine different families (21, 22). Our report described the presence of rare mutations and the predominance of AR types. Of note, this predominance of the AR types is in accordance with other Middle Eastern countries (16, 19, 21, 23, 24) in which consanguineous marriages prevail and is in contrast to other countries and regions, such as the United States of America, South America, Europe, China, and Japan, where the X-linked disease is exceedingly more prevalent than the AR types (4, 5, 17, 25–27).

Here, we present the second report of clinical and genetic characteristics of 31 CGD patients suffering mainly from AR-CGD. Only one case of X-CGD was reported. Twenty-two Jordanian, seven Lybian, and two Iraqi CGD patients from 21 different families were investigated. Most of the marriages were consanguineous except five. Most of the patients underwent extensive clinical and laboratory investigations to clarify the relationship between the identification of the mutated protein and the clinical severity in very rare AR-CGD forms affecting p22^*phox*^ and p67^*phox*^ mainly and p47^*phox*^ too, and in one case of X-CGD affecting NOX2. Eleven patients from the same families, who did not have laboratory but only clinical investigations, were suspected to have been died from CGD too. Because of high frequency of the c.1171_ 1175delAAGCT mutation found in *NCF2*, we report the first founder effect of this mutation in eight unrelated Jordan families by performing segregation analysis with nine polymorphic markers overlapping the *NCF2* gene.

## Patients and Methods

### Ethical Considerations

**Figure 1:**
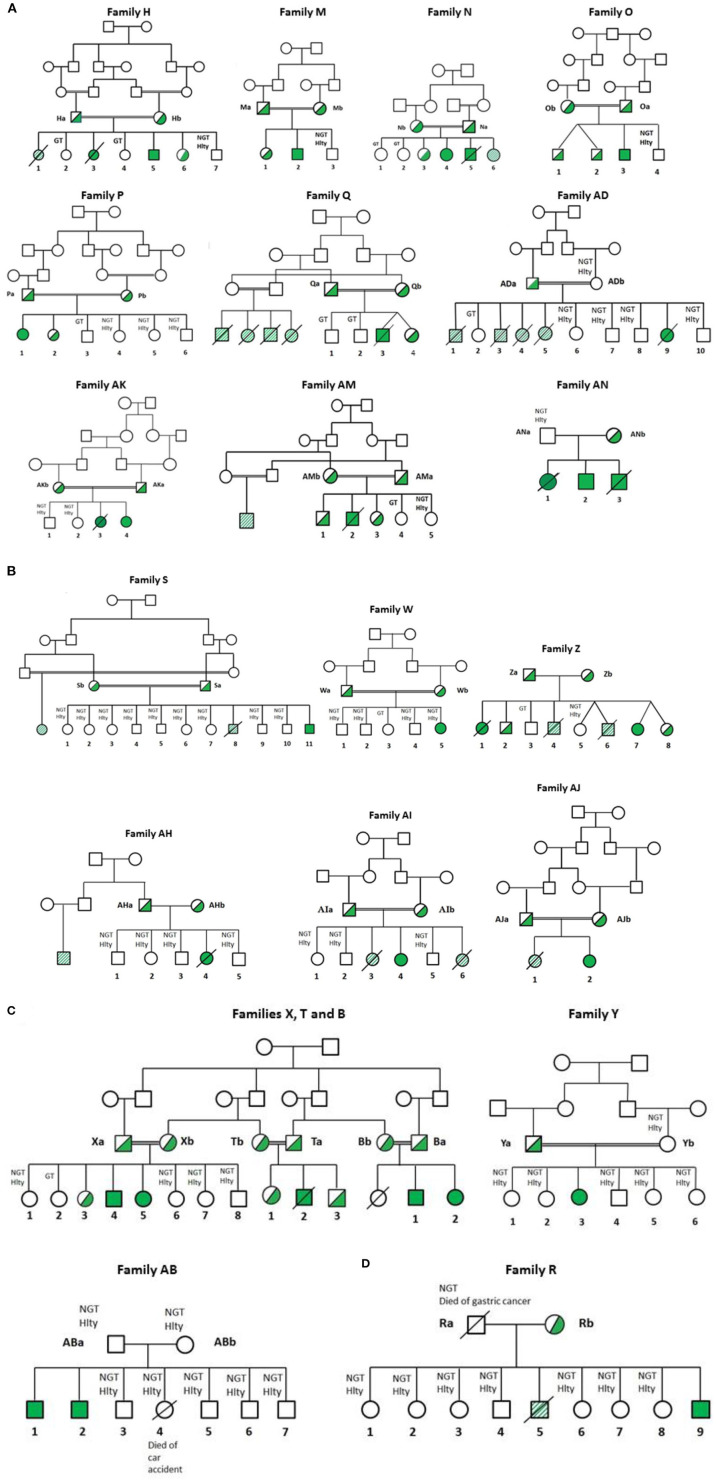
Pedigree of Jordanian, Lybian, and Iraqi families. CGD was diagnosed by measurement of NADPH oxidase activity in neutrophils as described in Patients and Methods. All families contained consanguineous marriages, except families R, Z, AB, AH, and AN. Diagonal shaded box: highly suspicious but functionally and genetically not tested (H1, R5, S8, Z6, Z4, AD1, AD3, AD4 and AD5, AI3 and AJ1). Hlty, healthy; NGT, not genetically tested; GT, genetically tested; Open symbols: no mutation in CGD genes among the tested members; filled symbols: homozygous for the CGD mutations; half-filled symbols: heterozygous for the mutation; cross-out symbols: deceased patients. **(A)** CGD patients from families H, M, N, O, P, Q, AK, AM, AN, and AD suffering from AR67^0^ CGD. **(B)** CGD patients from families S, W, Z, AH, AI, and AJ suffering from AR22^0^ CGD. **(C)** CGD patients from families T, Y, X, and AB suffering from AR47^0^ CGD. T and X are related families. These families were also related to family B (21). **(D)** CGD patients from family R suffering from X^0^ CGD.

Blood samples were collected from healthy volunteers, their patients and relatives after obtaining signed informed consent. Written consents for DNA analysis of samples from patients, their parents and relatives were also obtained. CGD patients were identified from personal communications with relevant specialist physicians during the study period (Jan 2006–Sep 2019). The patients with confirmed diagnosis (or suspected diagnosis) of CGD were referred to the main study center (Division of Infectious Diseases, Jordan University Hospital, Amman, Jordan) for further clinical investigations. Twenty-two Jordanian, seven Libyan, and two Iraqi CGD patients (17 females, 14 males) from 21 different families were investigated. All patients underwent extensive clinical (Jordan University Hospital in Amman, Jordan) and laboratory investigations (CGD Diagnosis and Research Centre, Grenoble, France). All patients underwent extensive clinical and laboratory investigations. Clinical history was taken from medical records and interviewing the families. In addition, we reviewed the clinical characteristics of 15 patients of the same set of families who did not have genetic testing but had either an abnormal Nitroblue Tetrazolium (NBT) test (for NADPH oxidase activity) or clinical suspicion of CGD. Below is a brief description of patients with emphasis on important complications. Clinical data and pedigrees are shown in Table 1 and Figure 1, respectively. All families were Jordanian unless otherwise specified. Follow-up was continued until November 2019 unless mentioned otherwise.

**Table 1:** Summary of clinical histories of CGD patients.

**N^**°**^**	**Patients**	**CGD type**	**Sex**	**Origin**	**Age**	**Diagnosis**	**Diagnosis: confirmed or suspected**	**Severe infections/ complications**	**Minor infections/complications**	**Treatment**
1	H5	AR67^0^	M	Jordan	13 years	3 years	Confirmed	Lung abscess requiring, lobectomy	Weakness, pallor	2 Allo BMT, doing very well
2	H3	AR67^0^	F	Jordan	2 and $\frac{1}{2}$ years^†^	2 and $\frac{1}{2}$ years	Confirmed	Pulmonary Aspergillosis, alveolar hemorrhage	Weakness, pallor, failure to thrive, hepatomegaly, iron deficiency anemia	Antibiotic, anti-TB, methylprednisolone, caspofungin
	*H1*	*AR67^0^?*	*F*	*Jordan*	*2 years*^†^	*ND*	*Suspected*	Ascites	Lymphadenitis, skin abscesses, fever	TMP/SMX and itraconazole
3	M2	AR67^0^	M	Jordan	12 years	2 and $\frac{1}{2}$ years	Confirmed	—	Recurrent cervical lymphadenitis	Cephalexin, itraconazole
4	N4	AR67^0^	F	Jordan	17 years	4 months	Confirmed	Fungal lung abscess and lobectomy	Discharging ulcer after the BCG vaccine, thrombocytopenia, dental abscess, failure to thrive	Ciprofloxacin, voriconazole and prednisolone; TMP/SMX or itraconazole intolerance
5	N5	AR67^0^	M	Jordan	9 years^†^	2 months	Confirmed	Pneumonia, aspergillosis	Failure to thrive, hepatosplenomegaly	TMP/SMX and itraconazole
6	N6	AR67^0^	F	Jordan	2 years	1 y and 2 months	Confirmed	Colonic ulcers	Diarrhea, thrombocytosis	TMP/SMX and itraconazole
7	O3	AR67^0^	M	Jordan	16 years	4 years	Confirmed	Severe eye infection, lung infection mostly fungal	Gluteal muscle abscess, Leukocytosis, recurrent diarrhea, discharging ulcer after the BCG vaccine, perianal abscess, peritonitis, cervical lymphadenopathy	TMP/SMX and itraconazole, anti-TB,Allo BMT; doing very well
8	P1	AR67^0^	F	Jordan	13 years	3 years	Confirmed	Sepsis, pneumonia	Fever, lymphadenitis, knee septic arthritis, febrile reactions to vaccines, failure to thrive	TMP/SMX and itraconazole
9	Q3	AR67^0^	M	Jordan	20 years^†^	8 months	Confirmed	Pneumonia, meningitis, hydrocephalus, peritonitis and perforated viscus.	Failure to thrive	Amoxicillin, itraconazole and prednisolone
10	R9	X91^0^	M	Jordan	17 years	2 years	Confirmed		Fever and urinary tract infections, failure to thrive	TMP/SMX
	*R5*	*X91^0^?*	*M*	*Jordan*	*10 years*^†^	–	*Suspected*	Pneumonia, splenectomy		TMP/SMX and itraconazole
11	S11	AR22^0^	M	Iraq	26 years (follow up lost in 2011)	17 years	confirmed	Liver abscesses	Cervical and axillary lymphadenitis, skin abscesses	Rifampicin (anti-TB), then TMP/SMX and itraconazole
	*S8*	*AR22^0^?*	*M*	*Iraq*	*10 years*^†^	*-*	*Suspected*	Severe BCG reaction, tuberculosis and hemoptysis		TMP/SMX and itraconazole
12	T2	AR47^0^	F	Jordan	9 years^†^	7 years	Confirmed	Lung abscesses, emphysema with subsequent lobectomy		Allo BMT (matched sibling donor with myeloablative conditioning)Died 35 days after bone marrow transplant
13	W5	AR22^0^	F	Libya	20 years	1 year	Confirmed	Pulmonary tuberculosis, respiratory tract infections, gastric ulcer with upper gastrointestinal bleeding	Perianal abscess, cervical lymphadenitis, fever, urinary tract infections	TMP/SMX and itraconazoleSuccessful allo BMT
14	X4	AR47^0^	M	Jordan	18 years	14 years	Confirmed	Tuberculosis	Anemia	Rifampicin (anti-TB) then TMP/SMX
15	X5	AR47^0^	F	Jordan	14 years	7 years	Confirmed	Large mass in the lung	Cutaneous leishmaniosis	TMP/SMX
16	Y3	AR47^0^	F	Jordan	27 years	2 years	Confirmed	Pulmonary aspergillosis (three episodes), hand osteomyelitis, sepsis;	Skin abscesses	BMT complicated with pancreatitis, haemorrhagic cystitis, vertebral stress fractures, Addisonian crisis; bronchiolitis organizing pneumonia obliterans, bronchiectasis TMP/SMX and ciprofloxacin
17	Z7 *(twin*)	AR22^0^	F	Libya	(Follow up was lost in 2013)	3 months	Confirmed		Fever, mild hepatosplenomegaly, cervical lymphadenitis, urinary tract infections, enlarged kidneys with prominent papillae	TMP/SMX and itraconazole
18	Z1	AR22^0^	F	Libya	8 years^†^	6 years	Confirmed	Osteomyelitis, chronic pneumonia and possible tuberculosis	Vesicular exanthema, lymphadenitis, failure to thrive	TMP/SMX and itraconazole
	*Z6*	*AR22^0^?*	*M*	*Libya*	*14 months*^†^	*-*	*Suspected*	Fatal febrile illness		TMP/SMX and itraconazole
	*Z4*	*AR22^0^?*	*M*	*Libya*	*7 months*^†^	*-*	*Suspected*	Fatal vaccine febrile illness		TMP/SMX and itraconazole
19	AB1	AR47^0^	M	Jordan	35 years	18 years	Confirmed	Recurrent pneumonia	Fever, recurrent bronchitis, oral ulcers, cervical lymphadenitis	Interferon γ, TMP/SMX
20	AB2	AR47^0^	M	Jordan	34 years	17 years	Confirmed	Recurrent pneumonia	Lymphadenitis, skin abscesses.	Interferon γ, TMP/SMX
21	AD9	AR67^0^	F	Iraq	8 years^†^	3 years	Confirmed	Recurrent pneumonia	Cervical lymphadenitis	Intravenous antibiotics
	*AD1*	*AR67^0^?*	*M*	Iraq	*2 years*^†^		*Suspected*	Pneumonia	Cervical lymphadenitis and skin abscesses	TMP/SMX and itraconazole
	*AD3*	*AR67^0^?*	*M*	*Iraq*	*6 months*^†^		*Suspected*	Pneumonia and seizures		TMP/SMX and itraconazole
	*AD4*	*AR67^0^?*	*F*	*Iraq*	*8 months*^†^		*Suspected*	Pneumonia		TMP/SMX and itraconazole
	*AD5*	*AR67^0^?*	*F*	*Iraq*	*9 years*^†^		*Suspected*	Tuberculosis, severe pneumonia and liver disease	Abscess at right lateral malleolus, infection following BCG vaccine, cervical lymphadenitis	anti-TB, TMP/SMX
22	AH4	AR22^0^	F	Libya	1 year^†^	3 months	Confirmed	severe pneumonia, died after BMT due to severe pneumonia	Weakness (excessive sleeping and poor sucking)	Intravenous antibiotics, BMT but died because of lung infection
23	AI4	AR22^0^	F	Libya	8 years	1 year	Confirmed	Recurrent pneumonia, liver abscess	Dental caries, BCG reaction, skin abscess	TMP/SMX and itraconazole
	*AI3*	*AR22^0^?*	*F*	*Libya*	*2 months*^†^		*Suspected*	Tachypnea	Fever	TMP/SMX and itraconazole
24	AI6	AR22^0^	F	Libya	1 year^†^	< 1 year	Confirmed	Recurrent pneumonia		TMP/SMX and itraconazole
25	AJ2	AR22^0^	F	Libya	6 years	6 months	Confirmed		Generalized lymphadenitis, failure to thrive	TMP/SMX and itraconazole
	*AJ1*	*AR22^0^?*	*F*	*Libya*	*3 years*^†^		*Suspected*	Recurrent pneumonia, bacteraemia (sepsis)	Sore throat	TMP/SMX and itraconazole
26	AK4	AR67^0^	F	Jordan	9 years	6 months	Confirmed	Pneumonia, blood stream infection (sepsis)	Left axillary abscess after BCG vaccine	Rifampicin, isoniazid (anti-TB), ethambutol, clarithromycin
27	AK3	AR67^0^	F	Jordan	5 years^†^	6 months	Confirmed	Recurrent pneumonia, blood stream infection (sepsis), brain infection	Axillary abscess after BCG vaccine	TMP/SMX and itraconazole
28	AM2	AR67^0^	M	Jordan	9 years^†^	9 years	Confirmed	Pneumonia	Left axillary abscess after BCG, cervical lymphadenitis	Intraveinous TMP/SMX and itraconazole
29	AN2	AR67^0^	M	Jordan	10 years^†^	10 years	Confirmed		Posterior cervical lymphadenitis	TMP/SMX and itraconazole
30	AN3	AR67^0^	M	Jordan	3 years^†^	3 years	Confirmed		Fever, weight loss, and small draining chest wall abscess	TMP/SMX and itraconazole
31	AN1	AR67^0^	F	Jordan	8 years^†^	6 years	Confirmed	Colitis, brain abscess, hip aspergillosis,	Severe anemia	TMP/SMX and itraconazole, Steroids,

? *means that CGD was suspected because the patient exhibited a clinical profile compatible with CGD, and there is a CGD patient in his family but no biological diagnosis was done before his death*.

† *Means that the patient died*. *Allo BMT, Allogenic Bone Marrow Transplantation; TMP/SMX, Trimethoprim/Sulfamethoxazole; anti-TB, antituberculosis treatment*.

### Cell Preparations

Blood samples were obtained from the CGD patients with appropriate institutional consent. Neutrophils were purified by Ficoll isolation and isotonic lysis of red blood cells (28). Epstein Barr virus-immortalized B-lymphocytes were obtained as described previously (29).

### Measurement of NADPH Oxidase Activity in Purified Human Neutrophils

For the CGD diagnosis, NADPH oxidase activity was measured in purified neutrophils using the classical test of reduction of NBT by superoxide (${\text{O}}_{2}^{-.}$) release from neutrophils during phagocytosis of opsonized latex particules (30). Superoxide was sometimes measured in an SOD-sensitive cytochrome ***c***reduction test (31). Reactive oxygen species produced by neutrophils after phorbol-myristate acetate (PMA) stimulation, were also measured by flow cytometry in the presence of dihydrorhodamine-1,2,3 (DHR) (20).

### Western Blotting

Expression of gp91^*phox*^ (NOX2), p22^*phox*^, p47^*phox*^, p67^*phox*^, and p40^*phox*^ in a 1% Triton X100 soluble extract prepared from human neutrophils was examined by Western blot analysis (50 μg of protein in each lane) (32) using monoclonal antibodies anti-NOX2 48 and anti-p22^*phox*^ 449 (33, 34) and polyclonal antibodies anti-p67^*phox*^ (C19, SC7662, Santa Cruz), anti-p47^*phox*^ and anti-p40^*phox*^ (35, 36). Polyclonal anti-IgG-HRP was used as a second antibody and the immune complexes were detected by chemiluminescence in an ECL kit Femtomax (Rockland Immunochemicals, Limerick, PA, USA). Protein concentration was determined by Pierce BCA Protein Assay^TM^ Kit (ThermoFisher, Illkirsh, France) (37).

### Analysis of the Expression of NADPH Oxidase Components by Flow Cytometry

Monoclonal antibody (mab) 7D5 directed against external epitopes of gp91^*phox*^ (NOX2) (D162-3, Clinisciences, Nanterre, France), monoclonal anti-p22^*phox*^ (SC-130550, Santa Cruz Biotechnologies Inc, Heidelberg, Germany), monoclonal anti-p67^*phox*^ (AB109523/EPR5065, Abcam, Paris, France) and monoclonal anti-p47^*phox*^ (AB179457/EPR 13134, Abcam, Paris, France) with secondary antibody conjugated with Alexa-Fluor 488 (A11070, Invitrogen Life technologies, Villebon sur Yvette, France) or PE (A10543, Invitrogen Life Technologies, Villebon sur Yvette, France), were used for analysis of NADPH oxidase subunit expression in phagocytic cells (2 × 10^5^ cells). In case of p22^*phox*^, p47 ^*phox*^, or p67 ^*phox*^ expression analysis, neutrophils were previously fixed in PFA 2% (v/v) and then incubated with specific antibodies directed against p22^*phox*^, p47^*phox*^, or p67^*phox*^ diluted in PBS, BSA 0.2% (w/v), saponin 0.01% (v/v) (38). Control staining with appropriate isotype-matched control antibodies was included to establish thresholds for positive staining. Cell fluorescence was quantified with a FACS Canto II (BD Biosciences). Data were collected and analyzed with the FACS DIVA software and FlowJo software-Tree Star (BD Biosciences, Pont de Claix, France).

### Molecular Analysis

#### Preparation of RNA and DNA

Total RNA was isolated from either mononuclear leukocytes or EBV-transformed B-lymphocytes of CGD patients and healthy individuals, using a modified single-step method (39). Genomic DNA was purified with a purification kit (ref 51206 Flexigene DNA kit, Qiagen, Hilden, Germany).

#### Sequencing

When possible, first-strand cDNA was synthesized from total RNA by reverse transcriptase reaction according to the manufacturer's instructions (ref 11EMAMV203, MP Biomedicals Santa Ana, CA, USA). Total cDNA was immediately amplified by PCR with appropriate primers and separated by gel electrophoresis. All PCR products were sequenced with an ABI 3730 XL 96 capillary sequencer (Perkin Elmer, Foster City, CA, USA). More than 500 samples of control cDNA of gp91^*phox*^ (NOX2), p22^*phox*^, p67^*phox*^, and p47^*phox*^ were analyzed to rule out the possibility of polymorphisms. In all cases, location of mutations found in cDNA was verified in genomic DNA after PCR amplification of each exon and flanking intron regions with appropriate forward and backward primers, followed by Sanger sequencing (21). Aliquots of all PCR products in bromophenol blue solution were run together with a DNA ladder (ref R0211, ThermoFisher Scientific, Illkirch, France) on 1.5% (wt/vol) agarose containing Gel Red^TM^ nucleic acid stain (ref 41003, Biotium, Inc, Fremont, CA, USA) in parallel with a negative control (PCR products amplified without DNA) and a positive control (PCR amplification of control DNA) to analyze size and purity. In some cases, PCR products were purified from agarose gel according to manufacturer instruction (QIAquick Gel Extraction kit, ref 28704, Qiagen, Courtaboeuf, France).

### Gene-Scan Method to Determine the Ration of *NCF1* and *NCF1* Pseudogenes (ψ*NCF1*)

Fragments of genomic DNA from AR47^0^ CGD patients and their relatives were amplified with primers that anneal with regions in *NCF1* as well as in **ψ***NCF1* regions around the GTGT sequence at the start of exon 2, with PCR conditions according to Dekker et al. (40). The mixture of *NCF1* and ψ*NCF1* products was analyzed in a sequencer ABI 3730 XL 96 capillary sequencer (Perkin Elmer, Foster City, CA, USA) to determine the ratio between the number of *NCF1* and ψ*NCF1* genes present (40, 41).

### Homozygosity Mapping With Microsatellites Markers and Estimation of the Most Recent Ancestor of *NCF2* Mutation

Homozygosity mapping was performed with genomic DNA from eight patients of eight different Jordan families (H, N, O, P, Q, AK, AM, and AN). We used a set of nine microsatellite markers (D1S212, D1S2751, D1S2640, D1S2619, D1S2623, D1S2701, D1S2711, D1S202, and D1S238) previously described (42). Amplification of the microsatellite markers were performed by multiplex PCR. Sizes were determined with Genemapper® Software. The estimation of the most recent ancestor (MRCA) was realized with the algorithm developed by Gandolfo et al. (43), which is based on the shared haplotypes length between patients with the same mutation.

## Results

### General Consideration

Clinical and genetic characteristics of CGD were analyzed in 22 Jordanian, seven Libyan and, two Iraqi patients from 21 families (Tables 1, 2); 14 families were from Jordan (H, M, N, O, P, Q, R, T, X, Y, AB, AK, AM, and AN), five from Libya (W, Z, AH, AI, and AJ), and two from Iraq (S and AD). All families were consanguineous except 5 (R, Z, AB, AH, and AN). Genetic results are presented from a total of 27 patients; 26 were tested in this study (H5, H3, M2, N4, N5, O3, P1, Q3, R9, S11, T2, W5, X4, X5, Y3, Z7, AB1, AB2, AD9, AH4, AI4, AJ2, AK4, AM2, AN2, and AN3) and one patient had abnormal NBT test and genetic testing in Germany before she died (Z1). NBT testing alone was performed in a total of 4 patients (N6, AI6, AK3, and AN1). Clinical suspicion for CGD was present in 11 patients (H1, R5, S8, Z4, Z6, AD1, AD3, AD4, AD5, AI3, and AJ1). The overall mortality among the tested families was 3/4 (75%) among those who had abnormal NBT but no genetic testing. The overall mortality was 11/11 (100%) among those who were only clinically suspected. Furthermore, there were seven affected relatives; two relatives with abnormal NBT test (a cousin in the AH family and a cousin in the AM family), five with clinical suspicion (four cousins in the Q family and a cousin in the S family). All patients suffered from AR-CGD except one patient in the R family who suffered from X-CGD. A relationship was established between families T and X. These families were also related to family B described in our previous article (21). The diagnosis of CGD was suspected on clinical grounds and confirmed by functional and genetic studies of the patients.

**Table 2:** Phenotype and genotype of CGD patients and their relatives.

**N^**°**^**	**Patients**	**NBT test *% positive cells***	**Cyt C reduction *nmol/min/10^**6**^cells***	DHR stimulation index^*^	**Western blot or flow cytometry**	**Nucleotide**	**Gene location**	**Amino-acid change**	**Protein domain**	**CGD sub-type**
1	H5 *m*	0	0	/	Abs p67*phox*	c.1171_1175delAAGCT	*NCF2*- Exon 12	p.Lys391Glufs^*^9	PB1 domain	AR67^0^
2	H3 *f*^†^	/	/	/	Abs p67*phox*	c.1171_1175delAAGCT	*NCF2*- Exon 12	p.Lys391Glufs^*^9	PB1 domain	AR67^0^
	*H1 f*^†^	*/*	*/*	*/*	*/*	*/*	*/*	*/*		*AR67^0^ ?*
	H2 *f (sister)*	93	14.8	/	Pres p67*phox*					Not carrier
	H4 *f (sister)*	94	13.1	/	Pres p67*phox*					Not carrier
	Father	95	13.9	/	Pres p67*phox*					Carrier
	Mother	96	15.6	/	Pres p67*phox*					Carrier
**3**	**M2** ***m***	**0**	**0**		**Abs p67*****phox***	**c.505 C>G and deletion of exon 6**	**c.609+372_670-655**	**p.Gln169Glu and del. Exon 6**	**Activation domain**	**AR67**^**0**^**, New mutation**
	M1 *f (sister)*	/	/	/	Pres p67*phox*					Carrier
	M3 *m (brother)*	98	/	/	Pres p67*phox*					/
	Father	/	3.4	/	Pres p67*phox*					Carrier
	Mother	/	4.2	/	Pres p67*phox*					Carrier
4	N4 *f*	0	0	/	Abs p67*phox*	c.1171_1175delAAGCT	*NCF2*- Exon 12	p.Lys391Glufs^*^9	PB1 domain	AR67^0^
5	N5 *m*^†^	/	0	/	Abs p67*phox*	c.1171_1175delAAGCT	*NCF2*- Exon 12	p.Lys391Glufs^*^9	PB1 domain	AR67^0^
6	N6 *f*	0	/	/	/	/	*/*	/	/	AR67^0^
	N1 *f (sister)*	/	/	/	Pres p67*phox*					Not carrier
	N2 *f (sister)*	/	/	/	Pres p67*phox*					Not carrier
	N3 *f (sister)*	/	/	/	Pres p67*phox*					Carrier
	Father	54	10.4	/	Pres p67*phox*					Carrier
	Mother	85	10.3	/	Pres p67*phox*					Carrier
7	O3 *m*	/	/	/	Abs p67*phox*	c.1171_1175delAAGCT	*NCF2*- Exon 12	p.Lys391Glufs^*^9	PB1 domain	AR67^0^
	O1 *m (twin)*	/	/	/	Pres p67*phox*					Carrier
	O2 *m (twin)*	/	/	/	Pres p67*phox*					Carrier
	Father	/	/	/	Pres p67*phox*					Carrier
	Mother	/	/	/	Pres p67*phox*					Carrier
8	P1 *f*	/	0	/	Abs p67*phox*	c.1171_1175delAAGCT	*NCF2*- Exon 12	p.Lys391Glufs^*^9	PB1 domain	AR67^0^
	P2 *f (sister)*	/	7.6	/	Pres p67*phox*					Carrier
	P3 *m (brother)*	/	/	/	Pres p67*phox*					Not carrier
	Father	/	4.8	/	Pres p67*phox*					Carrier
	Mother	/	/	/	Pres p67*phox*					Carrier
9	Q3 *m (twin)*	0	0	/	Abs p67*phox*	c.1171_1175delAAGCT	*NCF2*- Exon 12	p.Lys391Glufs^*^9	PB1 domain	AR67^0^
	Q1 *m (brother)*	91	5.7	/	Pres p67*phox*					Not carrier
	Q2 *m (brother)*	95	5.9	/	Pres p67*phox*					Not carrier
	Q4 *f (twin)*	90	5.7	/	Pres p67*phox*					Carrier
	Father	91	4.2	/	Pres p67*phox*					Carrier
	Mother	93	4.4	/	Pres p67*phox*					Carrier
10	R9 *m*		0		Abs NOX2	c252 G>T, del exon 3	*CYBB*- exon 3	p.Ser48_Ala84del	II-TM and B-Loop	X91^0^CGD
	*R5 m †*	/	/	/	/	/	*/*	/	/	*X91^0^CGD ?*
	*Mother*	/	3.8	/	Pres NOX2					Carrier
11	S11 *m*	/	0	/	Abs *p22phox*	c.268C>T	*CYBA*- exon 4	p. Arg90Trp	C-term	AR22^0^
	*S8 m*^†^	*/*	*/*	*/*	*/*	*/*	*/*	*/*	*/*	*AR22^0^ ?*
	Father	/	19.0	/	Pres p22*phox*					Carrier
	Mother	/	/	/	Pres p22*phox*					Carrier
12	T2 *m*	0	/	/	Abs p47*phox*	c.579G>A	*NCF1*- exon 7	p.Trp193^*^	SH3-1 domain	AR47^0^
	T1 *f (sister)*	95	/	/	Pres p47*phox*					Carrier
	T3 *m (brother)*	93	/	/	Pres p47*phox*					Carrier
	Father	95	/	/	Pres p47*phox*					Carrier
	Mother	98	/	/	Pres p47*phox*					Carrier
13	W5 *f*	/	/	/	Abs *p22phox*	c.295_301delGTGCCCG	*CYBA*- exon 5	p.Val99Profs^*^90	TM	AR22^0^
	W3 *f (sister)*	/	/	/	Pres p22*phox*					Not carrier
	Father	/	/	/	Pres p22*phox*					Carrier
	Mother	/	/	/	Pres p22*phox*					Carrier
14	X4 *m*	/	/	/	Abs p47*phox*	c.579G>A	*NCF1*- exon 7	p.Trp193^*^	SH3-1 domain	AR47^0^
15	X5 *f*	/	/	/	Abs p47*phox*	c.579G>A	*NCF1*- exon 7	p.Trp193^*^	SH3-1 domain	AR47^0^
	X2 *f (sister)*	/	/	/	Pres p47*phox*					Not carrier
	X3 *f (sister)*	/	/	/	Pres p47*phox*					Carrier
	Father	/	/	/	Pres p47*phox*					Carrier
	Mother	/	/	/	Pres p47*phox*					Carrier
16	Y3 *f*	/	/	/	Abs p47*phox*	c.75_76delGT	*NCF1*- exon 2	p.Tyr26Hisfs^*^26	PX domain	AR47^0^
	Father	/	/	/	Pres p47*phox*					Carrier
17	Z7 *f (twin)*	0	/	1.0	Abs *p22phox*	c.295_301delGTGCCCG	*CYBA*- exon 5	p.Val99Profs^*^90	TM	AR22^0^
18	Z1 *f* ^†^^$^	0	/	/	Abs p22*phox*	c.295_301delGTGCCCG	*CYBA*- exon 5	p.Val99Profs^*^90	TM	AR22^0^
	*Z6 m*^†^	/	/	/	*/*	*/*	*/*	*/*		*AR22^0^ ?*
	*Z4 m*^†^	/	/	/	*/*	*/*	*/*	*/*		*AR22^0^ ?*
	Z8 *f (twin)*	/	/	/	Pres p22*phox*					Carrier
	Z2 *m (brother)*	/	/	/	Pres p22*phox*					Carrier
	Z3 *m (brother)*	/	/	/	Pres p22*phox*					Not carrier
	Father	/	/	/	Pres p22*phox*					Carrier
	Mother	/	/	/	Pres p22*phox*					Carrier
19	AB1 *m*	**/**	/	1.3	Abs p47*phox*	c.579G>A	*NCF1*- exon 7	p.Trp193^*^	SH3-1 domain	AR47^0^
20	AB2 *m*	**/**	/	1.3	Abs p47*phox*	c.579G>A	*NCF1*- exon 7	p.Trp193^*^	SH3-1 domain	AR47^0^
21	AD9 *f*^†^	/	/	1.0	Abs *p67phox*	c.229C>T	*NCF2 - exon 2*	p.Arg77^*^	TPR domain	AR67^0^
	*AD1 m*^†^	/	/	/	/	/	*/*	/	/	*AR67^0^ ?*
	*AD3 m*^†^	/	/	/	/	/	*/*	/	/	*AR67^0^ ?*
	*AD4 f*^†^	/	/	/	/	/	*/*	/	/	*AR67^0^ ?*
	*AD5 f*^†^	/	/	/	/	/	*/*	/	/	*AR67^0^ ?*
	AD2 *f (sister)*	/	/	/	Pres p67*phox*					Not carrier
	Father	/	/	/	Pres p67*phox*					Carrier
22	AH4 *f*^†^	/	/	1.0	Abs p22*phox*	c.295_301delGTGCCCG	*CYBA*-exon 5	p.Val99Profs^*^90	TM	AR22^0^
	Father	/	/	/	Pres p22*phox*					Carrier
	Mother	/	/	/	Pres p22*phox*					Carrier
23	AI4 *f*	/	/	1.0	Abs p22*phox*	c.295_301delGTGCCCG	*CYBA*- exon 5	p.Val99Profs^*^90	TM	AR22^0^
24	AI6 f^†^	0	/	/	/	/	/	/	/	AR22^0^
	*AI3 f*^†^	/	/	/	/	/	*/*	/	/	*AR22^0^ ?*
	Father	/	/	35.4	Pres p22*phox*					Carrier
	Mother	/	/	21.3	Pres p22*phox*					Carrier
25	AJ2 *f*	/	/	1.0	Abs p22*phox*	c.295_301delGTGCCCG	*CYBA*- exon 5	p.Val99Profs^*^90	TM	AR22^0^
	*AJ1 f*	/	/	/	/	/	*/*	/	/	*AR22^0^ ?*
	Father	/	/	49.6	Pres p22*phox*					Carrier
	Mother	/	/	44.6	Pres p22*phox*					Carrier
26	AK4 *f*	/	/	/	Abs p67*phox*	c.1171_1175delAAGCT	*NCF2*- Exon 12	p.Lys391Glufs^*^9	PB1 domain	AR67^0^
27	AK3 *f*^†^	0	/	/	/	/	*/*	/	/	AR67^0^
	Father	/	/	/	Pres p67*phox*					Carrier
	Mother	/	/	/	Pres p67*phox*					Carrier
28	AM2 *m*^†^	/	/	1.0	Abs p67*phox*	c.1171_1175delAAGCT	*NCF2*- Exon 12	p.Lys391Glufs^*^9	PB1 domain	AR67^0^
	AM1*m (brother)*	/	/	/	Pres p67*phox*					Carrier
	AM3 *f (sister)*	/	/	/	Pres p67*phox*					Carrier
	AM4 *f (sister)*	/	/	/	Pres p67*phox*					Not carrier
	Father	/	/	/	Pres p67*phox*					Carrier
	Mother	/	/	/	Pres p67*phox*					Carrier
29	AN2 *m*	/	/	/	Abs p67*phox*	c.1171_1175delAAGCT	*NCF2*- Exon 12	p.Lys391Glufs^*^9	PB1 domain	AR67^0^
30	AN3 *m*^†^	/	/	/	Abs p67*phox*	c.1171_1175delAAGCT	*NCF2*- Exon 12	p.Lys391Glufs^*^9	PB1 domain	AR67^0^
31	AN1 *f*^†^	0	/	/	/	/	*/*	/	/	AR67^0^
	Mother	/	/	/	Pres p67*phox*					Carrier
	Normal values (*n* = 100)	90 ± 6	11.3 ± 3.3	>5 (98 ± 2)	/	/	*/*	/	/	/

* *Stimulation Index (SI): MFI of activated neutrophils/MFI of resting neutrophils*.

$ *Patient Z1 was diagnosed in Germany (NBT test and genetic test). We consider a patient to have CGD when he or she has been diagnosed by a NBT test and the clinical signs are suggestive of CGD (N6, AI6, AK3, and AN1)*.

*^?^Means that the patient exhibited a clinical profile compatible with CGD but no biological diagnosis was done before his death and he has a CGD patient in his family (H1, R5, S8, Z4, Z6, AD1, AD3, AD4, AD5, AI3, and AJ1)*.

† *means that the patient died; / means not determined; m, male; f, female; Normal values were obtained from 100 donors; Abs, absent; Pres, present; protein expression was performed by western blotting or by flow cytometry as described in Patients and Methods*.

*Blue for AR67 CGD, green for X91 CGD, pink for AR22 CGD and pale orange for AR47 CGD. New mutations are in bold*.

### Clinical Features of the CGD Patients

**Figure 2:**
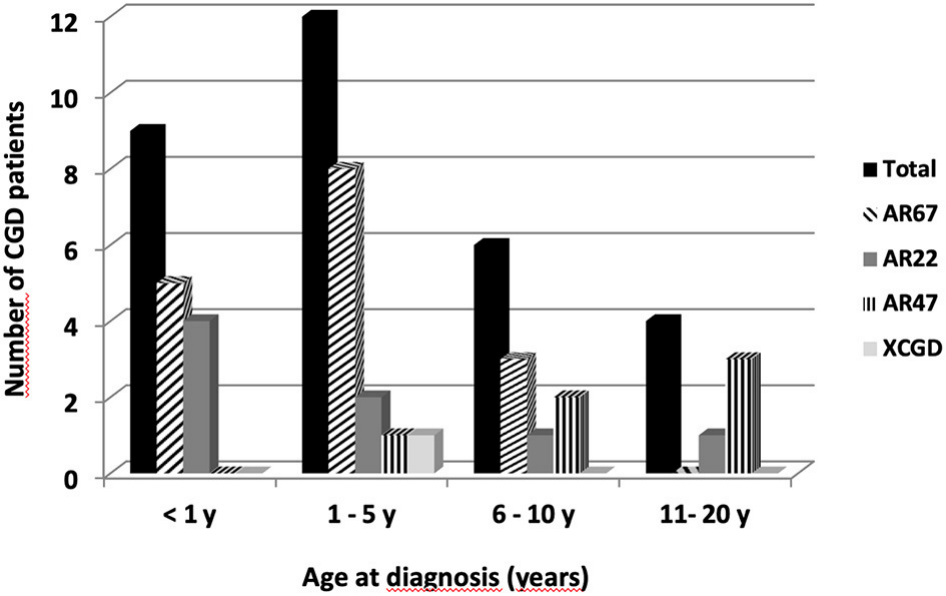
Age at diagnosis of the 31 CGD patients from Jordan, Iraq, and Lybian according to the CGD types. Twenty-two Jordanian, seven Lybian, and two Iraqi CGD patients from 21 different families were investigated. Sixteen patients suffered from AR67^0^ CGD, eight patients from AR22^0^ CGD, six from AR47^0^CGD, and only one patient had X-CGD.

Our analysis will focus mainly on comparing the different forms of AR-CGD because there is only one diagnosed case of X91^0^ CGD. One criterial aspect to judge the severity of this disease is to focus on the age of diagnosis (Figure 2). As can be seen AR67^0^ CGD and AR22^0^ CGD were diagnosed at the earliest time. Patients S11 and Z1 clinically suffered from AR22^0^ CGD at an early stage, although the diagnostic NBT test was done later. This is the same for patients AM2, AN1, and AN2 with AR67^0^ CGD (see Patients and Methods). On the other hand, the late age of diagnosis (18, 17, and 14 years) for patients with p47^*phox*^ deficiency (AB1, AB2, and X4) confirmed that this type of CGD is less severe than the other subtypes. Patient R9 who suffered from X91^0^ CGD was diagnosed early (at the age of 2, Table 1).

To analyze the relative severity of these different CGD forms, the diverse infections and complications of each patient were classified into severe or minor infections and complications (Table 3). The severe infections and complications are mainly located in the lungs, with pneumonia and lung abscesses by far the most common severe complications, regardless of the type of CGD (69 and 63% in AR67^0^ CGD and AR22^0^ CGD patients, respectively, and 100% in AR47^0^ CGD patients). The X91^0^ CGD patient R9 did not present any severe infections or complications although he was diagnosed at the age of 2 years. Until then, he had only growth retardation, anemia, and urinary tract infection, despite a total lack of NADPH oxidase activity (Table 2). However, his brother R5 had a splenectomy and died from pneumonia at the age of 10. Gastrointestinal complications occurred in approximately 13% of all AR-CGD patients, and sepsis was found also in the same proportion, except for AR22^0^ CGD patients who did not have sepsis (Table 3). Brain infections were found in 3 AR67^0^ CGD patients (Q3, AK3, and AN1), liver abscesses in 2 AR22^0^ CGD patients (S11 and AI4), osteomyelitis in one AR22^0^ CGD patient (Z1), and in one AR47^0^ CGD (Y3). Regarding the minor infections and complications, lymphadenitis and skin infections were by far the most common severe complications regardless of the type of AR-CGD (Table 3). Failure to thrive was also found relatively frequent in AR-CGD patients (38 and 25% in AR67^0^ CGD and AR22^0^ CGD patients, respectively). BCG vaccine reaction was also frequently observed in AR-CGD patients (31 and 13% in AR67^0^ CGD and in AR22^0^ CGD patients, respectively). Hepatomegaly was observed only in 2 AR67^0^ CGD patients (H3 and N5) and in 1 AR22^0^ CGD patient (Z7). Finally, urinary tract infections were rare and only observed in 2 AR22^0^ CGD patients (W5 and Z7). Other minor infections and complications were observed in only one or two patients (Table 3).

**Table 3:** Summary of the most common infections and complications in the CGD patients.

**Severe infections/complications**	**AR67**	**AR22**	**AR47**	**X91**	**Total**
	***n* = 16**	***n* = 8**	***n* = 6**	***n* = 1**	***n* = 31**
Pneumonia/Lung abcess	11 (69%)	5 (63%)	6 (100%)	/	22 (71%)
Gastrointestinal infection - Colitis	3 (19%)	1 (13%)	1 (17%)	/	5 (16%)
Sepsis	3 (19%)	/	1 (17%)	/	4 (13%)
Brain infection	3 (19%)	/	/	/	3 (10%)
Liver abscess	/	2 (25%)	/	/	2 (6%)
Osteomyelitis	/	1 (13%)	1 (17%)	/	2 (6%)
Eye infection	1 (6%)	/	/	/	1 (10%)
Hydrocephalus	1 (6%)	/	/	/	1 (10%)
**Minor infections/complications**					
Lymphadenitis	6 (38%)	5 (63%)	2 (33%)	/	13 (42%)
Skin abscess	2 (13%)	3 (38%)	3 (50%)	/	8 (26%)
Failure to thrive	6 (38%)	2 (25%)	/	1 (100%)	9 (29%)
BCG vaccine reaction	5 (31%)	1 (13%)	/	/	6 (19%)
Fever	2 (13%)	2 (25%)	1 (17%)	1 (100%)	6 (19%)
Anemia/thrombocytopenia/thrombocytosis	4 (25%)	/	1 (17%)	/	5 (16%)
Urinary tract infection	/	2 (25%)	/	1 (100%)	3 (10%)
Hepatosplenomegaly	2 (13%)	1 (13%)	/	/	3 (10%)
Weakness, palor	2 (13%)	1 (13%)	/	/	3 (10%)
Diarrhea	2 (13%)	/	/	/	2 (6%)
Dental abscess	1 (6%)	/	/	/	1 (3%)
Leukocytosis	1 (6%)	/	/	/	1 (3%)
Vesicular exanthema	/	1 (13%)	/	/	1 (3%)
Arthritis	1 (6%)	/	/	/	1 (3%)
Weight loss	1 (6%)	/	/	/	1 (3%)

Looking at the major severe complications suffered by the 11 potentially CGD patients who died before their laboratory diagnosis has been made, it is obvious that pneumonia was the most frequently occurring clinical event in AR-CGD patients (R5, AD1, AD3, AD4, AD5, AJ1). Sepsis (patients Z4, Z6, and AJ1), lymphadenitis (patients H1, AD1, and AD5), skin abscesses (patients H1 and AD1), and BCG vaccine reaction (patients AD5 and S8) were also frequently observed (Table 1).

Treatment of the CGD patients is outlined in Table 1. All patients had a prophylactic life-long treatment (most of the time TMP/SMX in combination with itraconazole). Patients H3, O3, S11, X4 AD5, and AK4 received in addition to TMP/SMX an anti-TB treatment to treat a suspected TB infection. When severe inflammatory syndrome was associated with infections, steroid therapy was given to patients H3, N4, Q3, and AN1. IFNγ was given to two patients for prophylaxis (AB1 and AB2). IFNγ was given to these two patients for only 1 year when it was available at that time. Six patients received allogeneic bone marrow transplantation (allo BMT) (H5, O3, W5, T2, Y3, and AH4). Three patients have complete chimerism and are doing well (H5, O3, W5). Patient T2 died 35 days after transplantation because of complications. Patient AH4 died after allo-BMT because of severe lung infection. Patient Y3 had severe complications such as pancreatitis, hemorrhagic cystitis, vertebral stress fractures and Addisonian crisis, but she is now maintained on TMP/SMX and ciprofloxacin.

To conclude from the AR-CGD patients' cohort studied, AR67^0^ CGD and AR22^0^ CGD appear to be equally severe clinical forms of the disease, whereas AR47^0^ CGD appears to have a milder clinical form. One exception is patient M2, who suffered from AR67^0^ CGD with mild clinical signs. Finally, our unique X91^0^ CGD patient suffered from a mild clinical form too, but this form cannot be considered as mild regarding the death of his brother from pneumonia at the age of 10.

### Analysis of the Molecular Base of the CGD Disease

**Figure 3:**
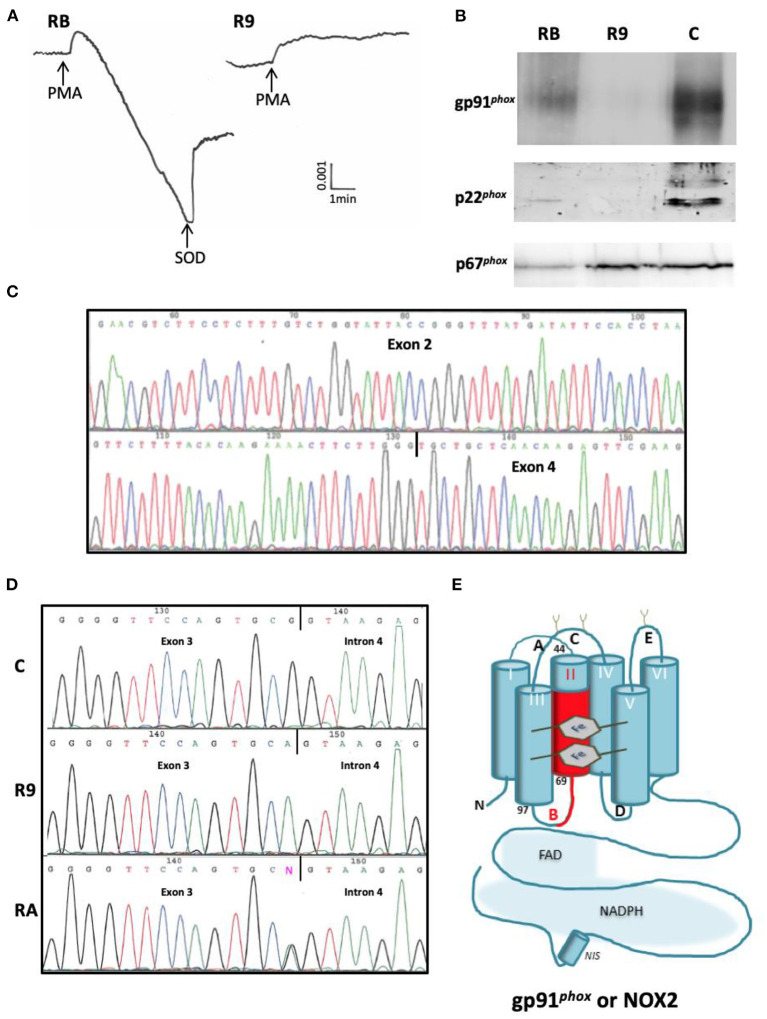
Phenotypic and genotypic characterization of the missense mutation c.252 G>T at the 3' end of exon 3 of *CYBB* in the X91^0^ CGD patient from family R. **(A)** NADPH oxidase activity measured by SOD sensitive-cytochrome *c* reduction with 5 × 10^5^ purified neutrophils stimulated by PMA (80 ng/mL). RB = mother, R9 = CGD patient. **(B)** Western-blot of p22^*phox*^ and gp91^*phox*^ analysis of soluble extracts (50 μg of protein in each lane) from purified neutrophils of Family R's members (RB mother, R9 CGD patient). The p67^*phox*^ expression was used to control the protein load. **(C)** Analysis of mutated p67^*phox*^ mRNA. **(D)** Analysis of mutated *NCF2* gene. **(E)** Consequence on the sequence of gp91^*phox*^ protein. The absence of exon 3 corresponds to the amino acid deletion p.Ser48_Ala84del in the second transmembrane domain of gp91^phox^ with a probable structural disorganization of the protein.

**X-CGD** with defects in the ***CYBB***gene was detected only in patient R9. Although its clinical form was not the most aggressive, we found no NADPH oxidase activity in his neutrophils. In his mother's neutrophils an intermediate value was measured (Figure 3A). No expression of gp91^*phox*^ and p22^*phox*^ in R9's neutrophils was detected (Figure 3B). This is understandable, since these two proteins need association for stable expression. His mother's neutrophils exhibited a diminished expression of both proteins. We amplified cDNA from gp91^*phox*^ mRNA in R9's leukocytes and found absence of exon 3 (confirmed by sequencing) in patient R9 (Figure 3C). Then exon 3 from the *CYBB* gene was sequenced and we found a silent hemizygous missense mutation c252 G>A at the end of exon 3, explaining skipping of exon 3 in the mRNA. The same mutation in one allele of *CYBB* was present in his mother, confirming her carrier status (Figure 3D). The mild clinical form of R9 cannot be explained by residual NADPH oxidase activity measured by cytochrome *c* reduction assay. In addition, his brother R5, suspected to have had CGD, died at the age of 10 years from pneumonia (Table 1).

**AR-CGD** with defects in the ***NCF1***gene were detected in 4 families (19%) T, X, Y, and AB and in six patients (19%) (T2, X4, X5, Y3, AB1, and AB2) (Table 2). Families T, X (related families), and AB exhibited the same mutation. Affected patients (T2, X4, X5, AB1, and AB2) were homozygous for the G579A mutation in codon Trp193 in exon 7 of *NCF1*. The parents of T2, his sister T1, and his brother T3 were all heterozygous for this mutation. The parents of X4 and X5, and their sister X3 were also heterozygous for this mutation. However, their sister X2 was unaffected. For all these patients CGD was discovered as a result of pneumonia (Tables 1, 3). The residual NADPH oxidase activity measured by flow cytometry (DHR probe) for patients AB1 and AB2 can explain the partial protection of these patients and the delay of the CGD diagnosis (Table 2). In addition, patients AB1 and AB2 are the oldest CGD patients of the cohort (34 and 35 years old, respectively) (Table 1). Unfortunately, the NADPH oxidase activity for patients T2, X4, and X5 was either not measured or measured by the NBT test, which is not sensitive enough to evaluate a residual NADPH oxidase activity, in contrast to flow cytometry (DHR). Patient Y3 exhibited the classical GT deletion (c.75_76delGT) in a GTGT repeat sequence in exon 2 of *NCF1*, which predicts a frameshift and a premature stop codon at residue 51. Her father was heterozygous for this mutation. We were not able to evaluate her mother. This mutation was also described in our previous report, in which all AR47^0^ CGD patients exhibited this mutation (21). The manifestation of the disease of patient Y3 was the most severe of all AR47^0^ CGD cases in this study, as she suffered from three episodes of lung infections, a sepsis and a hand osteomyelitis. In addition, she had severe complications after bone marrow transplant (BMT), but now she is maintained on TMP/SMX and ciprofloxacin. She's now 28 years old.

**AR-CGD** with defects in the ***CYBA***gene were detected in six families (19%) S, W, Z, AH, AI, and AJ and in eight patients (26%) (S11, W5, Z1, Z7, AH4, AI4, AI6, and AJ2) (Table 2). Patients W5, Z7, AH4, AI4, and AJ2 were all from Libya and showed a seven-basepair deletion c.295_301delGTGCCG in exon 5 of *CYBA*. This mutation was previously found in our first report (21). Patient AH4 died because of lung infection after BMT. Among these families, only the parents in family Z were not consanguineous. Patient Z1, and patients Z4, and Z6 from this family—not genetically tested—died, probably because they all suffered from AR22^0^ CGD. In addition, patients AI3, AI6 (NBT tested), and AJ1 (all not genetically tested) passed away too, confirming the severity of this AR22^0^ CGD type.

Patient S11 presented with a missense mutation c.268C>T in exon 4 of *CYBA*, changing Arg90 to Trp and leading to the absence of p22^*phox*^ expression. This mutation is rare and was reported only once, but was not functionally characterized (44). His brother S8 (not genetically tested) died at the age of 10 after a severe BCG reaction and hemoptysis. The NADPH oxidase activity, p22^*phox*^ and gp91^*phox*^ (NOX2) expression were abolished in the neutrophils of all AR22^0^ CGD patients (Table 2). This probably explains the severity of the clinical symptoms developed by these patients.

**Figure 4:**
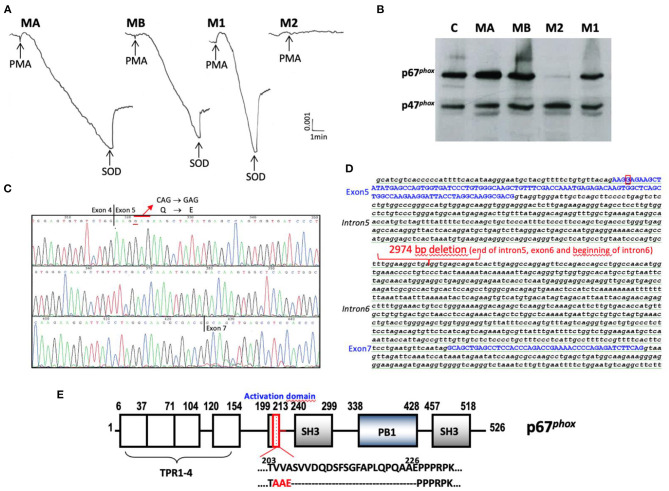
Phenotypic and genotypic characterization of the new mutation of the AR67^0^CGD patient from family M. **(A)** NADPH oxidase activity measured by SOD sensitive-cytochrome *c* reduction with 5 × 10^5^ purified neutrophils stimulated by PMA (80 ng/mL). **(B)** Western blot of cytosolic factors p47^*phox*^ and p67^*phox*^ analysis of soluble extracts (50 μg of protein in each lane) from purified neutrophils of family M (MA father, MB mother, M2 CGD patient, M1 sister). The p47^*phox*^ expression was used to control the protein load. **(C)** Analysis of mutated p67^*phox*^ mRNA. **(D)** Analysis of mutated *NCF2* gene. **(E)** Consequence on the sequence of p67^*phox*^ protein. The molecular consequence is an amino acid change p.ValValAla204-206AlaAlaGlu and p.Ser207-Glu226del in the activation domain of p67^*phox*^ protein that could be a potential interacting domain with NOX2.

**AR-CGD** with defects in the ***NCF2***gene were detected in 10 families (48%) H, M, N, O, P, Q, AD, AK, AM, and AN corresponding to 16 patients (52%) (H3, H5, M2, N4, N5, N6, O3, P1, Q3, AD9, AK3, AK4, AM2, AN1, AN2, and AN3) including 8 who died (H3, N5, Q3, AD9, AK3, AM2, AN1, and AN3) (Tables 1, 2). In addition, 7 patients (H1, AD1, AD3, AD4, AD5, AK3, and AN1) died probably because they suffered from AR67^0^ CGD but they were not genetically tested (N6, AK3, and AN1 diagnosed with an NBT test). These numerous deaths confirm that the AR67^0^ CGD is one of the most severe forms of the disease. Whenever possible, the NADPH oxidase activity of patients' neutrophils was measured and was shown to be totally abolished, as well as the expression of p67^*phox*^ (Table 2). An exception for disease severity may be made for patient M2 exhibiting a new mutation in *NCF2* (Figure 4). The NADPH oxidase measured in his parents' and sister's neutrophils was normal but null in the patient's neutrophils (cytochrome *c* reduction and NBT test) (Figure 4A, Table 2). Unfortunately, the NADPH oxidase activity could not be measured by the DHR oxidation in flow cytometry to detect a possible residual activity that could have explained the mild clinical form of this AR67^0^ CGD (Table 1). We found a very faint band of p67^*phox*^ by Western blotting in the M2 neutrophils (Figure 4B), and by amplifying the cDNA from the p67^*phox*^ mRNA, we found absence of exon 6 (confirmed by sequencing) (Figure 4C). We could not amplify exon 6 in the *NCF2* gene. After several attempts of amplification and sequencing we found a large deletion from part of the intron 5 to part of intron 6 (2,974 pb) (Figure 4D). His parents and his sister M1 were carriers of the mutation (Table 2). His brother M3 is in good health with a normal NBT test but his carrier status has not been determined.

Patient AD9 had a non-sense mutation in exon 2 of *NCF2* c.229C>T, changing the CGA codon for Arg77 into the TGA stop codon (p.Arg77^*^). This mutation was previously reported but is quite rare (45). The functional consequence of this mutation seems to be severe, because all patients (patients AD1, 3, 4, 5, and 9) from this family died, although AD1, 3, 4, and 5 were not genetically tested (Table 1). In addition, the NADPH oxidase activity measured in the neutrophils from patient AD9 by DHR oxidation in flow cytometry was null (Table 2). The father of these patients was carrier of the mutation whereas one of his daughters AD2 was unaffected. Unfortunately, the mother could not be tested.

**Figure 5:**
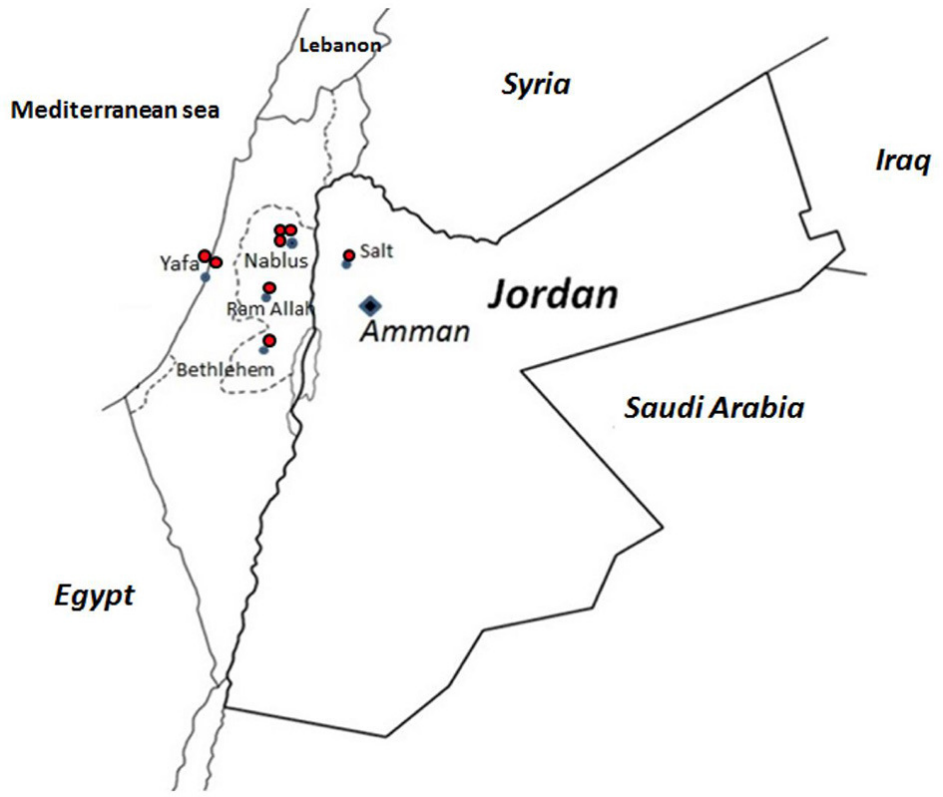
Geographical clustering of the Jordanian AR67 families sharing the c.1171_1175delAAGCT mutation in *NCF2*. The ancestor origin of each family is marked on the Jordan and Palestine map by a red dot.

All families (H, N, O, P, Q, AK, AM, and AN), except families M and AD, suffered from the same mutation, viz. a deletion of 5-bp AAGCT at position 1171 in exon 12 of the p67^*phox*^ cDNA (c.1171_1175delAAGCT). The molecular consequence is a frameshift starting at Lys391 and the introduction of a stop codon at Ser399 in the PB1 domain of p67*phox*. This mutation was reported previously (21, 46, 47). Like patient AD, all patients from these different families suffered from a severe clinical form of CGD (Table 1) with absence of NADPH oxidase activity and p67^*phox*^ expression (Table 2). Seven among 15 patients from these families died (Table 1). Because of the high frequency of this mutation in families H, N, O, P, Q, AK, AM, and AN, we decided to determine the founder effect of the c.1171_1175delAAGCT mutation using microsatellites markers to estimate the time of living of the most recent ancestor. Family AM was excluded from analysis since AM2 did not share any consecutive marker from the haplotype. The shared haplotype was estimated to 2.3 mega base between family H, N, O, P, Q, AK, and AN (Table 4). Assuming an independent genealogy the most recent common ancestor (MRCA) of the eight families was estimated to be 42.9 generations in the past [95% confidence intervals (CI): 21.9–63.5]. Assuming an independent genealogy, the MRCA of the eight families was estimated to have lived 37.2 generations ago (95% CI: 21.9– 63.5). Considering a generation time of 25 years, the common ancestor would have appeared 1,075 years ago (CI 550–1,600 years). A geographical clustering of the Jordanian AR67^0^ CGD families sharing the c.1171_1175delAAGCT mutation in *NCF2* can be seen in Figure 5.

**Table 4:** Haplotype study.

	**Microsatellite marker**	**D1S212**	**D1S2751**	**D1S2640**	**D1S2619**	**D1S2623**	**Mutation**	**D1S2701**	**D1S2711**	**D1S202**	**D1S238**
	**Chromosome position**	**178082616**	**180011174**	**180727202**	**182276183**	**182873602**	**Mutation**	**184622684**	**185417782**	**186865356**	**188146265**
Patient	H1	111/111	238/238	185/185	183/183	278/278	Homozygous	129/129	151/151	81/81	268/268
	N4	109/109	234/234	185/185	183/183	278/278	Homozygous	129/129	155/155	73/73	282/282
	O3	109/115	234/240	185/187	185/185	278/278	Homozygous	129/129	151/151	83/83	284/284
	P1	111/111	232/232	185/185	183/183	278/278	Homozygous	129/129	155/155	81/81	270/284
	AK4	109/109	238/238	185/185	183/183	278/278	Homozygous	129/129	151/151	81/81	270/270
	AM2	109/109	230/230	185/185	179/179	276/276	Homozygous	127/127	155/155	79/79	282/282
	AN2	107/107	238/238	197/197	183/183	278/278	Homozygous	129/129	151/151	83/83	280/280
	Q3	107/109	234/236	185/187	183/183	278/278	Homozygous	129/129	155/155	73/73	282/282

*Marker encompassing NCF2 with chromosome 1 position based on hg19 are shown. The founder haplotype is shaded in gray*.

## Discussion

The genetic analysis in the Arab cohort allows us to conclude that the major inheritance was AR with a predominance of AR67^0^ CGD (16 patients) over AR22^0^CGD (eight patients) and AR47^0^CGD (six patients). Overall, in the 31 patients, eight different mutations were found; three in *NCF2* including a new one, two in *CYBA*, two in *NCF1*, and 1 in *CYBB*. All these mutations were homozygous, indicating that both parents contributed an identical, mutated allele that caused the disease. Contrary to what is found in the Western population, only one male patient (R9) suffered from X-CGD, in a non-consanguineous family. This report confirms our previous finding that the most common form of CGD in Jordan is the AR type (21). The high frequency of AR-CGD appears also in Tunisia, Egypt, Turkey, Iran, Oman, Saudi Arabia, and Israel because of the high rate of consanguineous marriage in these countries (16, 19, 23, 24, 48–51) and it is in contrast to the published literature from USA, Europe, South America, China, and Japan, in which X-CGD is the main genetic form (4, 5, 17, 25–27, 52–54). The population in Jordan amounted from 5.3 million in 2004 to 10.2 million in 2020, and is composed of a variety of ethnic groups, the majority being Arabs. Indeed, many Arab countries display a long tradition of consanguinity due to socio-cultural factors. In Jordan 20–30% of all marriages occur between first cousins, and are strongly associated with the appearance of AR diseases (55, 56). This is also true for other Arab countries such as Iraq and Libya that are close to Jordan (57). In our study, only four families out of 14 Jordanian families (R, AB, AH, and AN) and one out of five Libyan families (Z) did not result from consanguineous marriages. The two Iraqi families (S and AD) were also consanguineous. No publication related to CGD in Iraq was found except in the Israeli cohort of 84 CGD patients comprising two Iraqi Jewish patients suffering from X-CGD (16).

The most frequent mutation found in *CYBA* (c.295_301delGTGCCCG) was found in 5 patients all originating from Libya (W, Z, AH, AI, and AJ families). This mutation was also found in five parents and relatives of these Libyan families who died. Whereas data on CGD from Libya are scarce (58, 59) one case of a child with the c.295_301delGTGCCCG mutation in *CYBA* has been described (60). Of note, this mutation was previously found in two patients from one Jordanian family (family J) and here in the Libyan patient AH4 (8, 21, 23, 44). In addition, this mutation was reported in one patient from a French cohort (without any nationality identification) (44) and in two patients out of 15 patients of a Tunisian cohort (23). This mutation was also present in 11 patients out of 28 Egyptian patients, highlighting that AR22^0^ CGD is the commonest form among the Egyptian population (49). Tunisia, Libya, and Egypt are three countries with common frontiers and not too far from Jordan. Our findings argue in favor of a possible founder effect since the single c.295_301 delGTGCCCG mutation in *CYBA* was detected mainly in patients from these countries. The molecular consequence of this mutation is the change of Val99 to Pro and the introduction of a stop codon in a potential transmembrane domain of p22^*phox*^, leading to the disorganization of the protein (21). The second mutation found in the Jordanian patient S11 is a missense mutation c.268C>T in exon 4 of *CYBA*, changing Arg90 to Trp and leading to the absence of p22^*phox*^ expression. This mutation was previously reported but not fully characterized (44). Arg90 is located in a potential transmembrane domain of p22^*phox*^ whose function is not very well-characterized but probably involved in the structural stability of the protein (61). AR22^0^ CGD with mutations c.268C>G, c.269G>A, and c.269G>C corresponding to the missense mutations Arg90Gly, Arg90Gln, and Arg90Pro in p22^*phox*^, respectively, were also previously reported, indicating the important structural role of this residue (8).

Six patients out of 31 from four Jordan families (T, Y, X, and AB) suffered from an AR47^0^ CGD. In patient Y3, the classical deletion (c.75_76delGT) in the GTGT repeat sequence at the beginning of exon 2 of *NCF1* gene leads to a frameshift and the introduction of a stop codon p.Tyr26Hisfs^*^26 in p47^*phox*^ protein. This mutation is located in the PX domain of p47^*phox*^ involved in the binding to phospholipids of the plasma membrane during the assembly of the activated NADPH oxidase complex (62, 63). This is a very common complication in CGD because of the presence of two pseudogenes close to the *NCF1* gene and crossing-over events in chromatin (7, 8). The most common AR form of CGD worldwide is caused by this mutation which represents 25% of total CGD cases and about 60% of all AR-CGD cases. In the first report of CGD characterization in Jordanian families, five patients out of 15 presented with this mutation (21). The c.75_76delGT mutation is also the most commonly reported *NCF1* mutation in Tunisia (23), Turkey (19), Egypt (49), Saudi Arabia (51), and India (64, 65). Then, the nonsense mutation c.579G>A in exon 7 of *NCF1* was found in five Jordan patients (families T, X, and AB). This mutation causes a change of the TGG codon for Trp193 into the TGA stop codon (41, 46, 66). It is situated in the first SH3 (SRC homology 3) domain of p47^*phox*^ involved in the binding with p22^*phox*^ during the NADPH oxidase assembly for activation (67). It is a quite worldwide rare mutation except in Israel (16, 46) and it is also the predominant *NCF1* mutation found in AR47^0^ CGD patients in Oman (48). In Israel population, the Trp193^*^ mutation is predominantly found in Kavkazi Jewish patients. The c.579G>A mutation in *NCF1* was introduced about 1,200–2,300 years ago in the Kavkazi Jewish population but was present in surrounding populations already for more than 5,000 years (68). Indeed, this mutation was found not only in Jordan (this report) but also in Turkish patients (19). In addition, the c.579G>A mutation in Kavkazi CGD patients is associated with a heterogeneous clinical phenotype (69).

The AR67^0^ CGD form was by far the most frequently found form of CGD found in the Jordan population. Mutations in *NCF2* were detected in nine out of 22 families, corresponding to 16 patients including eight deceased patients. All of the patients were from Jordan except patient AD9 who was from Iraq. He presented with a non-sense mutation in exon 2 of *NCF2*, leading to the introduction of a stop codon and predicting p.Arg77^*^. Two patients in a Turkish cohort of 89 CGD patients (19) and three patients in a Mexican cohort (17) exhibited the same mutation. The missense mutation Arg77Gln was reported too (70). Arg77 is localized in the tetratricopeptide (TPR) domain of p67^*phox*^, which is able to bind the G-protein rac2 during the activation process of the NADPH oxidase (45, 71). The functional consequence is a total absence of NADPH oxidase activity, which explains the severe phenotype found in family AD. Patient M2 exhibits a new deletion mutation in one allele of *NCF2* (Table 2). The molecular consequence is an amino acid change and a deletion in the activation domain of p67^*phox*^ protein, a potential interacting domain with gp91^*phox*^ (NOX2) (Figure 4E) (72). A missense mutation C.505 C>G was also found in the second allele of *NCF2*. This last mutation predicted the change p.Gln169Glu in p67^*phox*^ and has been found in both alleles of *NCF2*. However, the AR67 CGD subtype was not determined (8). He is the unique AR67^0^ CGD Jordanian patient suffering from a mild form of CGD. By Western blotting it seems that a faint band of p67^*phox*^ was present. At that time, it was not possible to measure the NADPH oxidase activity by flow cytometry (DHR), which is the proper means to detect residual activity that might explain his mild CGD clinical form. Besides these two rare *NCF2* mutations, eight Jordanian families (H, N, O, P, Q, AK, AM, and AN) with 11 CGD patients exhibited the same mutation c.1171_1175delAAGCT in exon 12 of *NCF2*, leading to a frameshift and the introduction of a stop codon p.Lys391Glufs^*^9 in the p67^*phox*^ protein. This mutation was the unique one found in all three families with a defect in *NCF2* described in our first report (21). It was also found in two patients from Jordan and Palestine (47). Indeed, geographical clustering of the AR67^0^ CGD Jordanian families sharing the c.1171_1175delAAGCT mutation in *NCF2* is in a region of Jordan close to Palestine. Assuming independent genealogy from eight Jordan families, we estimate the most recent common ancestor ~1,075 years ago between families sharing the haplotype. However, due to the absence of shared haplotype between the patient AM2 with the others, it is possible that the mutation is older. A founder effect of the c.2547+2T mutation in *NCF2* was also studied in 11 patients from six families in the West of Tunisia (42).

Finally, only one case of X-CGD was found here in the group of 31 CGD Jordanian patients. The splice mutation c.252G>T in exon 3 of *CYBB* as the origin of his disease was previously reported (9, 73). Skipping of exon 3 in gp91^*phox*^ cDNA correspond to the amino acid deletion p.Ser48_Ala84del in the second transmembrane domain of gp91^*phox*^ with a probable structural disorganization of the protein (Figure 3E). Kannengiesser et al. found no NADPH oxidase activity measured by the NBT test, chemiluminescence, and DCFH assays but did not analyze the gp91^*phox*^ (NOX2) expression in neutrophils (44). We also found no NADPH oxidase activity in the neutrophils of patient R9 measured by the SOD-sensitive cytochrome *c* reduction test. In addition, we did not find any gp91^*phox*^ (NOX2) and p22^*phox*^ expression by Western-blot analysis. This phenotype cannot explain the mild CGD clinical form observed in this patient but is in accordance with the severe clinical form of his brother R5 who died of pneumonia at the age of 10. Brunner et al. studied the functional impact of the c.252G>A mutation, which also induces the outsplicing of exon 3 (74). However, these authors showed that this mutation leads to the presence of few percent of normal mRNA next to the misspliced mRNA, explaining the residual NADPH oxidase activity which they measured in neutrophils from the patient by the DHR oxidation assay. Unfortunately, the authors did not measure gp91^*phox*^ (NOX2) protein expression. However, they explained the mild clinical form of the patient by the residual NADPH oxidase activity in his neutrophils (74).

A striking fact is that the forms most frequently found in Jordan and Libya are the AR67^0^ and AR22^0^ CGD. It is also the case in Egypt and Tunisia (23, 49). However, in Israel, Saudi Arabia, Libya, Oman, Iran, and India, the AR47^0^ CGD form prevails (16, 24, 48, 51, 59, 64, 65). However, in Turkey about 30–40% of the CGD patients have X-CGD, but AR47^0^ CGD represents about 30–50% of AR-CGD too (19, 75, 76). We have no clear explanation yet for this discrepancy apart from the appearance of ancestral mutations such as the c.1171_1175delAAGCT mutation in *NCF2* in Jordan and close countries and probably the same is true for the c.295_301 delGTGCCCG mutation in *CYBA*.

According to several clinical criteria, it appears clear that AR67^0^ CGD and AR22^0^CGD are by far the most severe clinical forms in the Jordanian CGD patients. AR47^0^ CGD appears to manifest much milder, except for the severe clinical form of AR47^0^ CGD in patient Y3 and the mild clinical form of AR67^0^ CGD in patient M2. Mild clinical CGD forms are certainly due to residual NADPH oxidase activity in CGD patients' neutrophils, as can be observed in AR47^0^ CGD (77) or “variants” of X-CGD such as X91^−^ CGD (18). The most sensitive method of measuring this activity is by far flow cytometry with the highly sensitive DHR fluorescent probe. Unfortunately, we were not always able to measure the NADPH oxidase activity by this method, but for sure this residual oxidant production may account for the better prognosis in AR47^0^ CGD, as in patients AB1 and AB2. Indeed, regarding the stimulation index (SI) of DHR oxidation values measured in the neutrophils of 89 CGD Turkish patients, CGD forms caused by mutations in *CYBA* and *NCF2* were as severe as the X^0^CGD subtype (19). This was previously observed in studies of large CGD patients' cohorts in Europe and USA (4, 5). In the Israeli experience with 84 CGD patients, the SI levels were significantly higher in the AR47^0^ CGD subtype with a better clinical score compared to other AR-CGD patients (16). However, the analysis of AR47^0^ CGD in the Kavkazi population reveals phenotypic heterogeneity in patients with the same *NCF1* mutation c.573G>A (69). Thus, we must remain cautious and bear in mind that other criteria influence the expression of the disease, such as early diagnosis, and how patient care and follow-up is provided for in referral centers.

## Data Availability Statement

The data that support the findings of this study are available on request from the corresponding author. The data are not publicly available due to privacy or ethical restrictions.

## Ethics Statement

The studies involving human participants were reviewed and approved by the Faculty of Medicine and deanship of research at The University of Jordan. Written informed consent to participate in this study was provided by the participants' legal guardian/next of kin.

## Author Contributions

FB did the clinical diagnosis, ensured the care and follow-up of patients and their families, collected the clinical data from all hospitals and medical centers that cared for CGD patients and their families, wrote the clinical description of the patients and their relatives, revised the manuscript, and drew Figures 1, 5 and Table 1. MM, SB, and BV performed the functional and molecular analysis. NR-B, JR, and JF performed the DNA sequencing. JR performed the segregating analysis. AA-W, RA, WH, AD, MEAS, MK, MMS, and JA-R did the clinical diagnosis, ensured the care and follow-up of patients and their families, and collected the clinical data. JF revised the manuscript. MJS carried out the conception, design of the study, acquisition, analysis, interpretation of data, drew Figures 2–4 and Table 2, and the drafting of the article. All authors contributed to the article and approved the submitted version.

## Conflict of Interest

The authors declare that the research was conducted in the absence of any commercial or financial relationships that could be construed as a potential conflict of interest.
