# Supplementary material for: Second Report of Chronic Granulomatous Disease in Jordan: Clinical and Genetic Description of 31 Patients From 21 Different Families, Including Families From Lybia and Iraq
Source: Front Immunol. 2021 Mar 5;12:639226. doi: 10.3389/fimmu.2021.639226 (PMC7973097; doi:10.3389/fimmu.2021.639226)
Supplement: Supplementary file 1 [file Data_Sheet_1.docx]

Supplementary Material

**Detailed clinical description**

***Family H***

Patient H5 was born in Aug 2006 from consanguineous parents (Figure 1A). He started to have weakness, pallor at age 2 years and 2 months. He later developed chest abscess requiring lobectomy. He was diagnosed with CGD at age 3 years. Soon after, bone marrow transplant was performed twice because the first transplant was not successful. He is alive and doing very well. Patient H3 was born in July 2003 (Figure 1A). She presented at age 2 years with fever for 2 months. She was found to have bilateral pulmonary infiltrate, failure to thrive, hepatomegaly, iron deficiency anemia, and hypergammaglobulinemia. Despite antibiotic and anti-TB therapy she remained febrile with leukocytosis ranging from 20,000/mL to 40,000/mL. A culture from her bronchoalveolar lavage grew *Aspergillus fumigatus*. Lung biopsy (performed on day 22) showed diffuse alveolar hemorrhage, and methylprednisolone was added between days 30 to 41. The diagnosis of CGD was made on day 44 by an NBT test. Subsequently, caspofungin was started. However, she died on day 46 from septic shock while being on the ventilator. Patient H1 had lymphadenitis and skin abscesses since birth. She died at age 2 years with fever and ascites. The CGD diagnosis was not confirmed for her by any test (Figure 1A).

***Family M***

Patient M2 was born in April 2007 from consanguineous parents (Figure 1A). He started to develop several episodes of cervical lymphadenitis requiring incision at age 2 years. Subsequently, he was diagnosed with CGD by NBT test at age of 2 years and a half. Since diagnosis, he is being maintained on prophylactic cephalexin and itraconazole without any complications. He is doing very well without any further illnesses or hospitalizations. His sister M1 and brother M3 are in good health.

***Family N***

Patient N4 was born in Dec 2002 from consanguineous parents (Figure 1A). She was diagnosed with CGD at age 4 months following the development of fungal chest abscess that required lobectomy. At age 5 years, she had severe thrombocytopenia that was treated with steroids. At age 8 years, she developed dental abscesses. She is on ciprofloxacin and voriconazole prophylaxis as she cannot tolerate Trimethoprim/Sulfamethoxazole (TMP/SMX) or itraconazole. She is also on prednisolone. Patient N5 was born in October 2004. He was diagnosed at age of 2 months following the development of pneumonia. He had two more severe episodes of pneumonia at age 2 and 9 years. The latter died due to aspergillosis infection in Jan 2013. Patient N6 was born in 2017. She had diarrhea, severe thrombocytosis, and colonic ulcers. She was diagnosed with CGD by an abnormal NBT at age 1 year and 2 months (Figure 1A).

***Family O***

Patient O3 was born in November 2003 from consanguineous parents (Figure 1A). He had unexplained leukocytosis at birth. His first infection was a severe eye infection at age of 1 month, then he began to have recurrent diarrhea with several hospital admissions. He developed a prolonged reaction to the BCG vaccine with discharging ulcer that took one year to heal. At 7 months, he had perianal abscess. At 4 years, he had peritonitis that responded only to antifungal therapy. At the same age, he developed cervical lymphadenopathy after which he was diagnosed with CGD by NBT testing. The lymphadenopathy failed to respond to antibacterial and antifungal medications but responded to steroids and was given TMP/SMX and itraconazole and later anti-TB therapy. He did well till age 10 years when he had gluteal muscle abscess and lung infection mostly fungal in origin, the abscess was treated by drainage. He underwent bone marrow transplantation at age 14. He is doing very well at present time.

***Family P***

Patient P1 was born in April 2006 from consanguineous parents (Figure 1A). At age 2 months, she started to have fungal skin infections and high fever reaching 40^0^ C following vaccinations. This was followed by skin abscesses mostly in the cervical area requiring surgical drainage and recurrent common cold and influenza like illnesses. At age 3 years, she was diagnosed with CGD by a negative NBT test following an attack of cervical lymphadenitis and sepsis. Since then, she had recurrent bronchitis, fever, and failure to thrive. At age 7 years she had right knee swelling, pain, fever that responded to course of antibiotics for several weeks. At age 11, she developed pneumonia. Currently she is on TMP/SMX and itraconazole.

***Family Q***

Patient Q3 was born in May 1999 from consanguineous parents (Figure 1A). At age 9 days he developed pneumonia then at age 1 month he developed meningitis. Thereafter, he had two episodes of pneumonia and three episodes of meningitis resulting in hydrocephalus with placement of ventriculo-vesical shunt. He has failure to thrive and was on itraconazole, amoxicillin, and prednisolone. At age 21, he developed severe peritonitis from a perforated viscus and died subsequently. The patient has four dead first degree cousins from a single family; two boys and two sisters. They all died of infections during childhood. The father of that family was the brother of the father of Q3 and the mother was the sister of the mother of Q3.

***Family S***

Patient S11 is from Iraq. He was born in July 1993 from consanguineous parents (Figure 1B). His first infection appeared at age 4 months with cervical and axillary lymphadenitis treated by rifampin for 1 and a half year with good improvement. He again had fever and lymphadenopathy at age 4 years for which he was treated for 9 months. At age 10 years, he developed skin abscesses all over his body including the face. At age 13, he had liver abscesses which recurred at age 17 years after which he was diagnosed with CGD by NBT testing. He is on prophylactic antibiotics. He had a brother (S8) who was suspected of having TB following BCG vaccination early in life and later died at age 10 years with hemoptysis that lasted for 3 days (Figure 1B). He also has a living cousin who has recurrent infections. Follow up was lost after the interview in 2011.

***Family T***

Patient T2 was born in Sept 2003 from consanguineous parents (Figure 1C). He was doing well till age 7 years when developed fever, fatigue, sweating and jaundice. He was and found to have lung abscesses and empyema for which he underwent lobectomy. During this time, he was diagnosed with CGD by an NBT test. Two years later he underwent bone marrow transplant which was complicated and resulted in patient’s death 35 days after transplant. He is related to family B (21) and family X (Figure 1C).

***Family R***

Patient R9 was born in Feb 2002 from non-consanguineous parents (Figure 1D). After birth, he started to have fever and urinary tract infections, and was diagnosed at age of 2 years with CGD by an NBT test. He has failure to thrive and is compliant on TMP/SMX prophylactic therapy. The father died of intestinal tumor. Patient R5 was born in 1986. At age 6 years, he began to have several attacks of severe respiratory tract infection. He also had splenectomy. He died at age 10 years and was never tested for CGD.

***Family W***

Patient W5 is from Libya. She was born in Dec 1999 from consanguineous parents (Figure 1B). The patient initially had perianal abscess at age 4 months followed by cervical lymphadenitis and was diagnosed with CGD at age 1 year by an NBT test. Later, she was diagnosed with pulmonary TB and was treated for one year. She also had fever, urinary and respiratory tracts infections, gastric ulcer with upper gastrointestinal bleeding, and occasional fever. She is adherent to TMP/SMX and itraconazole therapy. She underwent bone marrow transplant in Feb 2012 and is currently in good condition.

***Family X***

Patient X4 was born in Jan 2002 from consanguineous parents (Figure 1C). At age 6 years, he suffered from fatigue and anemia and was treated for TB for 18 months. An initial burst test at age 6 years was negative for CGD but a repeat test at age 14 years was suggestive of CGD. The patient is maintained on TMP/SMX and itraconazole and is doing very well. Patient X5 was born in Jan 2005 (Figure 1C). She had cutaneous leishmaniasis at age 2 years. The patient was diagnosed with CGD after developing left subclavian lymphadenopathy at age 7 years. A subsequent chest x ray revealed a large mass in the left lung which resolved with medical therapy. She is maintained on TMP/SMX and is doing very well. She is related to family B (21) and family T (Figure 1C).

***Family Y***

Patient Y3 was born in Feb. 1992 from consanguineous parents (Figure 1C). She began to develop skin abscesses at age 1 month at hands and axilla which required surgical drainage and resulted in osteomyelitis in one of her hands. At age 2 years she was diagnosed with CGD following an admission for sepsis with the detection of splenomegaly. Subsequently, she was treated with prophylactic TMP/SMX. She developed pulmonary aspergillosis three times at age 7, 11, and 12 years. The patient had bone marrow transplant at age 16 which was complicated by pancreatitis, hemorrhagic cystitis, vertebral stress fractures, and Addisonian crisis. At age 18 years, she developed bronchiolitis organizing pneumonia obliterans. Her most recent chest CT shows mild fibrosis and bronchiectasis. She is maintained on TMP/SMX and ciprofloxacin.

***Family Z***

Patient Z1 is from Libya. She was born in 2003 from non-consanguineous parents (Figure 1B). After birth, she developed staphylococcal vesicular rash. She later on suffered from osteomyelitis, lymphadenitis, and local skin infections after venipunctures, chronic pneumonia, and failure to thrive. At age 6 years, she was seen in a hospital in Berlin, Germany and was diagnosed with TB and AR22^0^CGD. At that time, she had elevated IgG to 2006 mg/dL (Normal, 590-1430 mg/dL), IgA 655 mg/dL (38-251 mg/dL), IgG1 1393 mg/dL (300-840 mg/dL), IgG2 297 mg/dL (70-255 mg/dL), IgG3 110 mg/dL (17-97 mg/dL). She had anemia, leukocytosis, and thrombocytosis. The patient died at age 8 years. A brother, Z4, was born in 2010 and died at age 7 months after 3 days of receiving a vaccine and subsequent febrile illness. Another brother, Z6, was born in 2011 and died at age 1 year and 2 months after a febrile illness that lasted for one month. Patient Z7. She was born in November 2011. She had fever and lymphadenitis at age 2 months and found to have mild hepatosplenomegaly, enlarged kidneys with prominent papillae. She also had urinary tract infection due to E coli. She was diagnosed with CGD by an abnormal NBT test at age 9 months. Follow up was lost with this family after the interview.

***Family AB***

Patient AB1 was born in Feb 1984 from non-consanguineous parents (Figure 1C). He initially developed prolonged febrile illness at age 5 years followed by recurrent bronchitis and pneumonia, oral ulcers, and cervical lymphadenitis (once at age 19 years). The diagnosis of CGD was made at age 18 years. He received Interferon γ following his diagnosis for one year. He is compliant with TMP/SMX. Patient AB2 was born in Dec 1985 (Figure 1C). He started to have recurrent pneumonia at age 4 years. At age 15, he had recurrent lymphadenitis and skin abscesses in the neck, axilla, and abdomen requiring surgical drainage. Diagnosis was made at age 17 years. He received Interferon therapy following his diagnosis for one year. He takes TMP/SMX intermittently.

***Family AD***

Patient AD9 is from Iraq. She was born in May 2011 from consanguineous parents (Figure 1A). At age 8 months, she started to have cervical lymphadenitis that required intravenous antibiotics and surgical drainage. Then she developed recurrent pneumonia. She was diagnosed with CGD by NBT test at age 3 years. At age 4, she had to go to a camp after an armed conflict where she was exposed to harsh weather. She developed fever for one week and died. Patient AD1 was born in 1994. At age 6 months, he developed cervical lymphadenitis and skin abscesses. At age 2, he developed severe pneumonia and subsequently died. Patient AD3 was born in 1997. He developed pneumonia and seizures. He died at age of 6 months. Patient AD4 was born in 1998. She died of pneumonia at age 8 months. Patient AD5 was born in 2001. Initially, she had infection following BCG vaccine. At age 6 months, she developed cervical lymphadenitis. She was given anti-TB therapy twice, each course for 6 months. She was given TMP-SMX at age 5 years empirically. At age 9, she developed severe pneumonia and liver disease and subsequently died. Patients AD1, AD3, AD4, and AD5 were not tested for CGD (Figure 1A).

***Family AH***

Patient AH4 is from Libya. She was born in Oct 2014 from non-consanguineous parents (Figure 1B). During the 7^th^ month of her gestational age, she was found to be weak and her delivery was 20 days late from the expected date of delivery. At age one month, she had excessive sleeping and poor sucking. At age 45 days, she had abscess at right lateral malleolus that required incision and one month of intravenous antibiotics. She was diagnosed with CGD at age of 3 months. At age 1 year, she developed severe lung infection. She underwent bone marrow transplantation after recovery but died 2 days after the transplant because of lung infection. She had a cousin (the son of the father's brother) who was diagnosed with CGD and died at age of 8 months.

***Family AI***

Patient AI4 is from Lybia. She was born in Aug 2011 from consanguineous parents (Figure 1B). At age 2 and half months, she developed an infection at site of BCG vaccine and was suspected to have CGD. She was diagnosed at age 1 year with CGD in Greece. Subsequently, she suffered from dental caries, recurrent pneumonia, liver abscess, and skin abscess requiring incision and drainage. The patient had 2 sisters: patient AI3, who died at age 2 months because of fever; and patient AI6, who was diagnosed with CGD and who died at age 1 year from recurrent pneumonia. Follow up was lost with this family after the interview (Figure 1B).

***Family AJ***

Patient AJ2 is from Libya. She was born in Nov 2013 from consanguineous parents (Figure 1A). At age 40 days, she developed fever and generalized lymphadenitis which was treated with antibiotics. She also suffered from failure to thrive. She was diagnosed with CGD at age 6 months. She has a sister AJ1 who at age 1 year had recurrent sore throat, bacteremia. At age 3, she developed pneumonia and eventually died of it. Follow up was lost with this family after the interview.

***Family AK***

Patient AK4 was born in 2010 from consanguineous parents (Figure 1A). She was diagnosed with CGD at age 6 months after developing left axillary abscess following BCG vaccination. This was managed by rifampin, isoniazid, ethambutol, and clarithromycin for one year. During that time, she suffered pneumonia and blood stream infection. Patient AK3 was born in 2008. She developed axillary abscess after BCG vaccination that required surgical drainage. She was diagnosed with CGD at age 6 months by NBT test. She developed recurrent pneumonia and blood stream infection. Eventually she died at age 5 years and a half of lung infection with brain involvement.

***Family AM***

Patient AM2 was born in October 2008 from consanguineous parents (Figure 1A). He developed left axillary abscess following BCG vaccination which required surgical drainage. Soon after, he developed cervical lymphadenitis for which he took intravenous antibiotics and subsequently recovered. He did well till age 8 years when he developed pneumonia and was hospitalized for 1 month. Shortly after this, he suffered another attack of severe pneumonia and was diagnosed at that time with CGD by an NBT test at age 9 years. He died soon after that. He has a first-degree male cousin who was diagnosed with CGD by an NBT test at age 1 year after BCG vaccination and after knowing the diagnosis of patient AM2.

***Family AN***

Patient AN1 was born in February 2015 from non-consanguineous parents (Figure 1A). At age 4 years, she had blood in stool and subsequently was diagnosed with Crohn's disease and was treated with steroids with good response. At age 5 years and a half, she developed right hip pain which was followed by fever and severe anemia with hemoglobin of 5 g/dL. Three months after this attack, the patient developed headache and was found to have brain abscesses and a subsequent hip biopsy showed aspergillosis. Following these events, she was diagnosed with CGD by an NBT test at age 6 years. She remained in stable condition until age 8 years when she again developed severe anemia (hemoglobin of 5 g/dL) and persistent fever. She died during this last disease episode and just after arriving from an air flight. Patient AN2 was born in 2009. At age 4 years, he developed posterior cervical lymphadenitis. At age 7 years, he again developed purulent cervical lymphadenitis. He had lymph node biopsy to rule out malignancy (Figure 1A). Patient AN3 was born in March 2016. At age one year and 8 months, he suffered from fever for one month. At age 2 years he had weight loss and small draining chest wall abscess. He was later found to have lung abscesses and subsequently died at age 2 years.
